# Influences and Mechanisms of Nano-C-S-H Gel Addition on Fresh Properties of the Cement-Based Materials with Sucrose as Retarder

**DOI:** 10.3390/ma13102345

**Published:** 2020-05-20

**Authors:** Deyu Kong, Guangpeng He, Haiwen Pan, Yuehui Weng, Ning Du, Jiansong Sheng

**Affiliations:** 1 College of Civil Engineering, Zhejiang University of Technology, Hangzhou 310023, China; 2111706353@zjut.edu.cn (G.H.); panhaiw1026@163.com (H.P.); 2111706045@zjut.edu.cn (Y.W.); 2111706042@zjut.edu.cn (N.D.); 2 Taizhou Branch, Zhejiang-California International Nanosystems Institute, Taizhou 318000, China; shengjiansong@126.com

**Keywords:** nano-C-S-H gel, sucrose, cement hydration, hydroxide, Ettringite

## Abstract

Influences and mechanisms of chemically synthesized nano-C-S-H gel addition on fresh properties of the cement-based materials with sucrose as a retarder were investigated in this study. The results showed that the flow value of the fresh cement paste was gradually but slightly reduced with increasing nano-C-S-H gel addition due to its fibrous but well-dispersed characteristic in both water and cement paste. The semi-adiabatic calorimetry testing results verified that incorporation of nano-C-S-H gel could greatly mitigate the retarding effect of sucrose on cement hydration. The total organic carbon (TOC) indicated that the addition of the nano-C-S-H gel helps to reduce adsorption of the sucrose molecules into the protective layer, thus the semi-permeability of the protective layer was less reduced and that is why the addition of the nano-C-S-H gel can mitigate the retardation caused by the sucrose. Through XRD analysis, it was found that the CH crystals are more prone to grow along the (0001) plane with larger size in the paste with nano-C-S-H addition before the induction period starts, because the C-S-H nanoparticles can form 3D network to slow down the diffusion rate of the released ions and eliminate the convection in the paste, thus suppress the 3D nucleation and growth of the CH crystals. The XRD analysis also indicated a refinement of the ettringite crystals in the paste with sucrose addition, but introduction of nano-C-S-H gel did not show further refinement, which was also verified by the SEM observation.

## 1. Introduction

As is well-known, the durability of steel and reinforced concrete structures served in the marine environment faces severe challenges due to the large amounts of corrosive ions such as Cl^−^, Na^+^ and SO_4_^2−^ in seawater. Since the high alkalinity in concrete can protect the reinforcing bars from corrosion, reinforced concrete structures are dominant in large-scale marine structures and coastal buildings. In recent decades, various novel materials and technologies have been used to improve corrosion resistance of the steel rebar and the concrete itself in reinforced concrete structures with prolonged designed service life, such as the epoxy coated rebar instead of conventional rebar to protect against corrosion of the reinforcement itself [1,2], the controlled permeable formwork instead of conventional impermeable formwork to remove excess water from cast concrete and improve the impermeability of the hardened concrete surface [3,4,5,6], the hydrophobic silane [7,8,9,10,11] or organic protective coating [12] for surface-treatment on concrete surface to inhibit chloride ions penetration, and so on. Apart from these additional measurements, the most direct, economical and effective way to protect the steel rebar from corrosion is to use high performance concrete (HPC) with excellent water and chloride ion penetration resistance and ensure appropriate thickness of the concrete protective layer covered on the steel rebar in the reinforced concrete structures [13].

While preparing HPC, reactive mineral admixtures including silica fume, slag, fly ash, etc., have been widely used to improve properties, mainly workability of the fresh concrete and durability of the hardened concrete [14,15]. Due to their pozzolanic reactivity, calcium hydroxide (CH), one of the main cement hydration product, which is easy to dissolve and react with corrosive ions to cause deterioration, can be consumed to produce pozzolanic calcium silicate hydrate (C-S-H gels) with higher strength and better stability, helping to improve microstructure of the hardened cement paste (HCP) and the interfacial transition zone (ITZ) between the aggregate and the HCP, thus the strength and the impermeability of the hardened concrete at the later curing ages can be greatly enhanced [16]. At the same time, the ultrafine particles in the mineral admixtures are supposed to not only function as fillers to release free water in the gap of the cement particles to improve workability of the fresh concrete and microstructure of the hardened concrete, but also as nucleation sites for precipitation of C-S-H gels from cement hydration to accelerate cement hydration and cause pore as well as grain refinement of matrix and ITZ based on the well-known through-solution mechanism for the C-S-H gel formation during cement hydration [16,17,18].

In recent years, numerous studies have focused on using nanoscale materials to further improve properties of the hardened concrete with and without mineral admixtures [19]. It was proposed that the nano-SiO_2_ with much higher pozzolanic reactivity than silica fume could act as fillers, pozzolan and seeds much more effectively than the traditional pozzolanic materials [20,21,22]. For those nanomaterials without pozzolanic reactivity, such as nano-TiO_2_ [23,24,25], nano-CaCO_3_ [26,27,28,29], nano-Al_2_O_3_ [30,31], nano-C-S-H [18,32,33,34], nano-clay [35,36,37], carbon nanotube [38,39] etc., they can also play important roles of filling and seeding effects to enhance mechanical and durable properties. Among these nanoscale materials, nano-C-S-H gel may be the earliest one, which was used as nucleating seeds to improve service life of the structures in actual projects [32]. The studies on field concrete revealed significant increases in durability and service life of the reinforced concrete where it was utilized [32].

On the other hand, retarding admixtures are often used in combination with superplasticizer to increase workability of fresh HPC, allowing for longer haul, placement, and finishing times [40,41], especially at high temperatures [41]. The retarding admixtures can be found in both organic and inorganic compounds, for example, the saccharides, the lignosulfonic acids and their salts, the hydroxy-carboxylic acids and their salts, the inorganic salts such as borates, phosphates, zinc and lead salts, etc. [41]. Among these, sucrose may be one of the most well-known strong retarders, which has been widely investigated in several decades [42,43,44,45,46,47,48]. However, Thomas et al. [18] found that the retarding effects of sucrose can be effectively mitigated and even negated if enough nano-C-S-H particles were incorporated at the time of mixing or reversed if they were added later after the retarding effect has already been established. Therefore, the effect of nano-C-S-H gel on properties of the fresh and hardened cement-based materials should be considered when they were incorporated together into the high-performance concrete used in the marine environment. Nevertheless, few studies about this can be found in the literature.

In addition, on the basis of the through-solution mechanism, it was generally proposed that sucrose retards cement hydration by adsorbing onto anhydrous or (more likely) the partially hydrated mineral surfaces, preventing further nucleation and growth through poisoning nucleation sites for C-S-H gels [42,43,44,45,46,47], and the addition of nucleation sites in the form of C-S-H seed can reduce or eliminate the retarding effect by providing an alternate pathway for the hydration process [18]. However, our previous studies argued whether nanoparticles incorporated in the cement-based materials could act as nucleation sites or not for C-S-H growth during cement hydration [49,50,51,52,53]. Through modeling experiments, no nucleus function can be observed for C-S-H growth in the hydrating system with nano-silica and nano-TiO_2_ addition [53]. It was proposed to more reasonably explain the observations by using the topochemical reaction rather than the through-solution mechanism for the C-S-H formation during cement hydration [53]. If this is true, then the retarding mechanism of the sucrose on cement hydration and the mitigating mechanism of the nano-C-S-H gel on retardation effect of the sucrose on cement hydration may need to be re-examined.

In this paper, influences of the nano-C-S-H incorporation on fluidity and cement hydration of the fresh cement-based materials with 0.2 wt.% sucrose as the retarder were investigated in details. By incorporating 0.2 wt.% sucrose in the cement paste, it was found that the cement hydration can be retarded greatly without affecting the fluidity of the fresh cement paste according to our trial tests. The mechanisms of their influences on the properties were analyzed and discussed through TOC measurement, XRD and SEM observation. It is believed that the knowledge gained through this investigation will be helpful to promote the usage of nano-C-S-H gel to further enhance the durability of the high-performance concrete with retarders, and also further elucidate the mechanisms of cement hydration.

## 2. Experimental Programs

### 2.1. Materials

**Figure 1:**
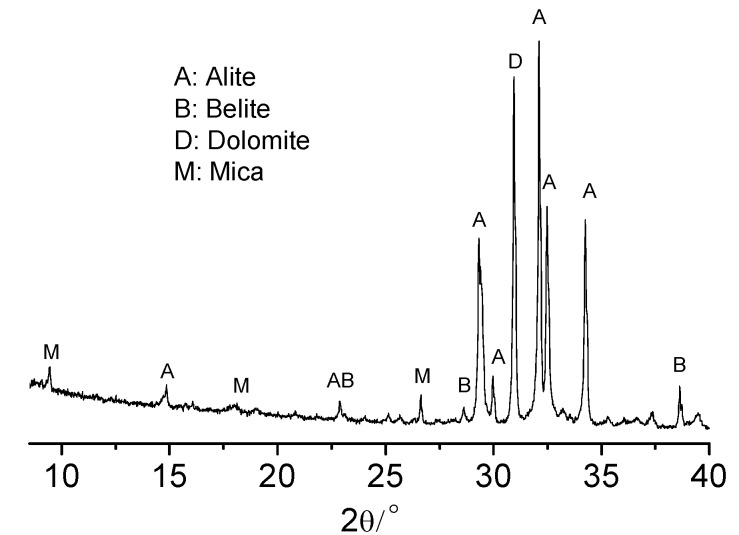
XRD diagram of the white cement.

Ordinary white Portland cement with strength grade of 52.5 purchased from Aalborg Portland (Anqing) Co. Ltd. (Anqing, China) was used in the study. The chemical composition, the physical and mechanical properties are shown in Table 1 and Table 2. The XRF (Table 1, IntellipowerTM 4200, Thermo Fisher Scientific, Waltham, MA, US, Rh 4GN, 4.2kW) and XRD (Figure 1, X’Pert PRO, PNAlytical, Almelo, Netherlands, CuKa radiation, 40mA, 40kV scan step: 1/60°, scan speed: 10°/min) analysis indicated that a small amount of dolomite containing some mica (the peak at about 9.45° of 2θ) was added as mineral admixture in the white cement. In the study a table sugar (sucrose) was used as the strong retarder.

**Figure 2:**
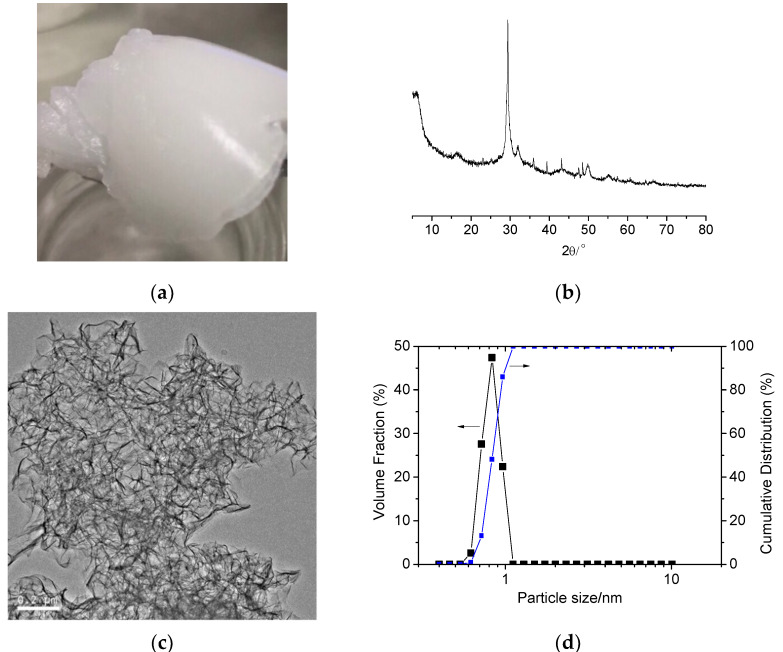
Characterization of the chemically synthesized C-S-H nanoparticles. (**a**) The gel; (**b**) XRD diagram; (**c**) TEM photograph and (**d**) particle size distribution.

The nano-C-S-H gel used in the study was prepared by precipitating from aqueous solutions of calcium chloride and sodium silicate (water–solid ratio of both solution is 30) at a Ca/Si molar ratio of 1:1. The solutions were prepared by using reagent-grade Na_2_SiO_3_·9H_2_O and anhydrous CaCl_2_ respectively. After aging for at least one day, the two solutions were mixed by adding sodium silicate solution into calcium chloride solution, causing immediate precipitation of the nano-C-S-H gel. After the mixture was shaken for 5 min and aging for one day, a Buchner funnel was used to filter the C-S-H precipitate while simultaneously rinsing with deionized water to remove sodium and chloride ions. During filtering and rinsing, the total dissolved solids (TDS) or the total amount of mobile charged ions in the filtrate were monitored by using a digital handheld water quality tester (TDS-3, Longsheng Hope Electronics Co. Ltd, Qingdao, China) and the total charged ion concentration of the filtrate was controlled below 500 ppm. After rinsing, filtering was continued until no more liquid could be removed, resulting in a translucent gel, see Figure 2a. The content of the solid C-S-H in the precipitate was measured to be about 5.26 wt.% by weighing the filtered precipitate before and after oven drying at 115 °C. Figure 2b presents the XRD pattern of the precipitated C-S-H gels. Its main diffraction peak is consistent with the diffraction peak of C-S-H. Figure 2c illustrates the TEM photograph of the C-S-H nanoparticles. As seen in Figure 2c, the C-S-H gels were fibrous in the nanoscale. The diameter of the nanoparticles is about several nanometers and the length can be as long as over 200 nm. From the TEM photograph, it seemed that the C-S-H nanoparticles also showed aggregation. However, the nanoparticle size of the C-S-H gels detected by using a laser analyzer (Nano ZS90, Malvern Panalytical, Malvern, England, dispersed in water) were in the range of 0.5365–1.117 nm, as presented in Figure 2d. It revealed that the nanoparticles in the C-S-H gels could be dispersed in water very well. From Figure 2d, it seemed that only the diameter of the nanoparticles can, but the length of them cannot, be detected by using the laser analyzer.

### 2.2. Methods

#### 2.2.1. Fluidity of the Fresh Cement Paste

**Figure 3:**
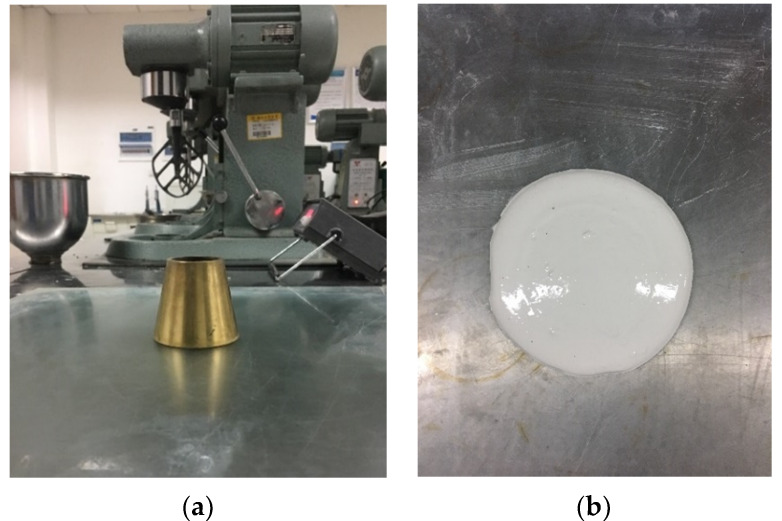
Fluidity testing of the fresh cement paste. (**a**) The mold used and (**b**) the paste spread.

The fluidity of the cement paste with 0.2 wt.% sucrose as retarder and also with nano-C-S-H gel was evaluated by the flow value of the paste filled in a truncated cone mold (36.0 mm × 60.0 mm × 60.0 mm) after the mold was lifted, as shown in Figure 3. Two values in the vertical direction were detected and the average was taken as the flow value of the fresh cement paste. The free water cement ratio of the fresh cement paste was fixed to be 0.40, in which the water contained in the C-S-H gels was included in the free water content.

#### 2.2.2. Hydration Heat

**Figure 4:**
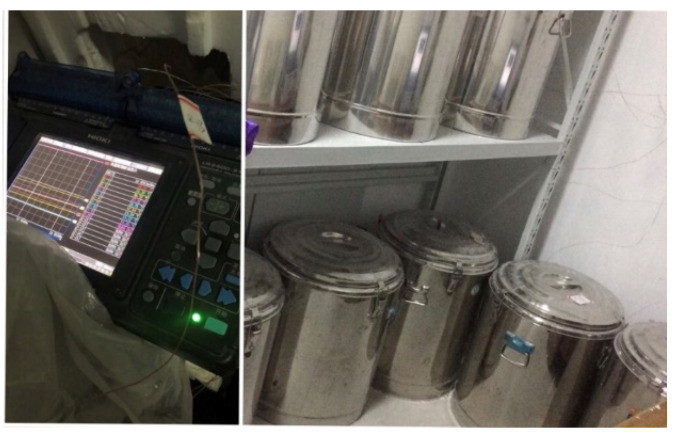
The semi-adiabatic calorimetry.

**Figure 5:**
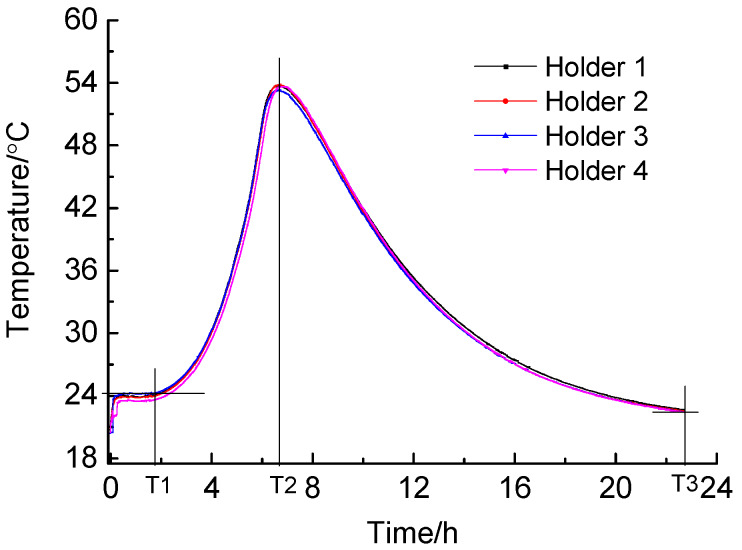
The consistency of the testing results for the blank sample in different insulative holders.

A kind of self-made semi-adiabatic calorimeter (Figure 4) was used to evaluate the effect of sucrose and nano-C-S-H gel on cement hydration. Pastes with 40 mL water and 100 g cement were prepared through manual mixing in a high speed for 60 s in a cylindrical plastic mold with an inner diameter of 47 mm and a height of 97 mm, in order to avoid the heat loss during the mixing process of the paste and in the period of transferring the paste from the steel pan to the testing mold if an automatic mixer is used. For the paste with nano-C-S-H addition, the C-S-H gel was introduced in water and the final water content for all tests was controlled to be nearly the same. After the paste was prepared, the mold was immediately placed in an insulative holder and a single Type J thermocouple inserted into the center of the paste. The temperature was then monitored by using the data-logger (HIOKI-LR8400-21, Hitachi (Shanghai) Trading Company, Shanghai, China) until the deceleration period ends. For the blank paste without sucrose and nano-C-S-H gel addition, eight samples were detected at the same time to check consistency of the testing results by using eight different insulative holders. Among these, the results detected by using four of them showed a very good consistency (Figure 5). Therefore, each time four samples were tested by using these four insulative holders and the environmental temperature was detected at the same time.

#### 2.2.3. Total Organic Carbon (TOC) Analysis

In this study, TOC analysis was used to detect the total organic carbon content in the solution filtered from the sucrose solution with nano-C-S-H gel addition or from the cement pastes with sucrose and nano-C-S-H gel addition to investigate the absorption of sucrose by the nano-C-S-H gel or by hydration products. While detecting the absorption of sucrose by nano-C-S-H gel, the sucrose solution with 0.2 g sucrose dissolved in 36.2 g water was prepared. Then 4.0 g of the nano-C-S-H gel was added into the sucrose solution. After shaking for 1 min and left settling without interference for 5 min, 10 min, 15 min, 30 min, 60 min and 120 min, the solution was filtered by using 0.45 μm membrane and then the filtrate was detected by using a TOC analyzer (Analytik Jena AG, Jena, Germany) to obtain the TOC content.

**Figure 6:**
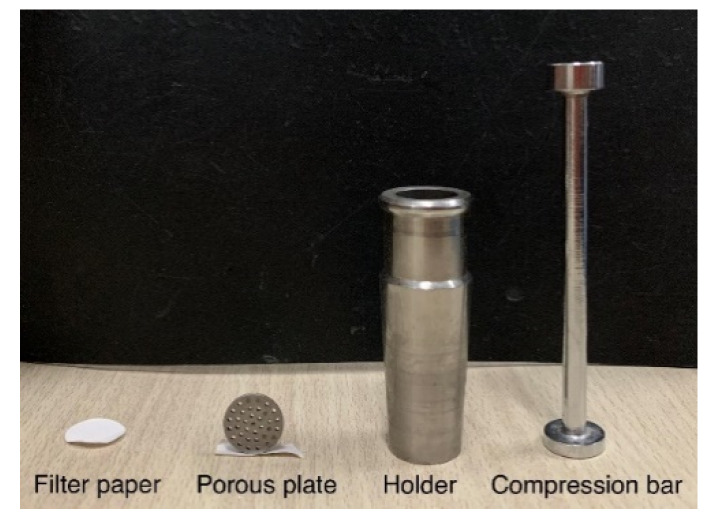
Setup for pressure-filtering of the fresh cement paste to obtain the solution for total organic carbon (TOC) testing.

While preparing the cement pastes with only 0.2 wt.% sucrose, or with both 0.2 wt.% sucrose and 4.0 wt.% nano-C-S-H gel, the sucrose and the nano-C-S-H gel were mixed into water to prepare the sucrose solution with and without nano-C-S-H gel addition firstly, then the cement was added into the sucrose solution and mixed for 3 min. The pastes were then cured at standard curing condition and the pore solutions of the fresh cement pastes were obtained through pressure-filtering at several time intervals until the initial setting by using a setup shown in Figure 6. The pore solution was then analyzed by using the TOC analyzer and the TOC content was estimated based on three replicate tests.

#### 2.2.4. Microstructure Investigation

Influence of nano-C-S-H gel addition on hydration products and microstructure of HCP with sucrose as a retarder was studied through XRD analysis and SEM observation. The pastes were prepared with the same water cement ratio as above. The additions of sucrose and nano-C-S-H were 0.2 wt.% and 4.0 wt.% respectively. For all samples with and without nano-C-S-H addition, the water content was kept nearly the same. The samples used for XRD analysis were taken at three time points, i.e., the time when the induction period ends and the acceleration starts (T1), the time when the exothermic peak appears (T2) and the time when the deceleration ends (T3) in the temperature rise curves during cement hydration, respectively, as indicated in Figure 5. For each sample, the fresh paste was cured in the semi-adiabatic calorimetric container until the time when the sample should be taken out as scheduled. For the samples at T1, they were directly washed twice with acetone and dried at 105 ± 5 °C for 24 h after they were taken out. For the samples at T2 and T3, they were washed twice with acetone, dried at 105 ± 5 °C for 24 h. The dried samples were then crushed, ground and then scanned by using the same XRD diffractometer under the same condition as shown above. The samples at T2 and T3 were also prepared for SEM observation. The samples were crushed and immersed in acetone for 3 days to terminate the hydration. After the samples were dried at 105 ± 5 °C for 24 h, the microstructure of the samples was observed by using SEM (HITACHI, S-4700, Hitachi, Tokyo, Japan).

## 3. Results and Discussions

### 3.1. Fluidity of the Fresh Cement Pastes

**Figure 7:**
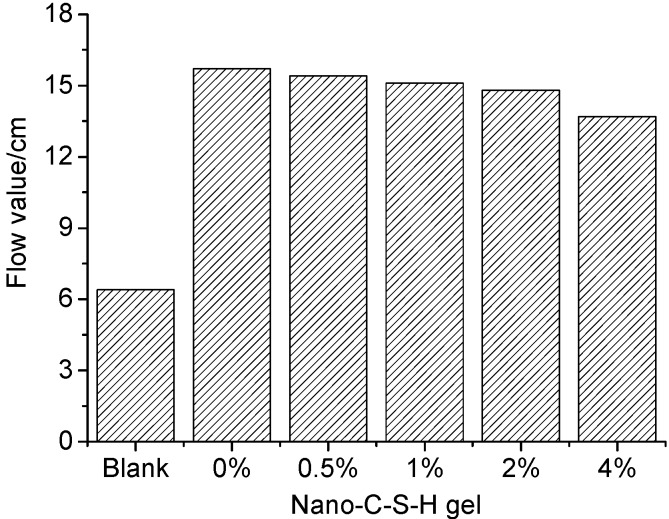
Effect of the nano-C-S-H gel on the fluidity of the fresh cement paste with 0.2 wt.% sucrose as the retarder.

Figure 7 shows the effect of nano-C-S-H addition on fluidity of the fresh cement mortar with sucrose as the retarder. As seen in Figure 7, the cement paste with 0.2 wt.% sucrose as the retarder showed an obvious increase in fluidity as compared to the blank paste due to the water-reducing effect of the sucrose on cement paste. The flow value was about 64.0 mm and 157.0 mm respectively for the blank paste and for the paste with 0.2 wt.% sucrose addition. When the nano C-S-H gel was further incorporated into the paste with 0.2 wt.% sucrose addition, the flow value tended to decrease slightly with increasing nano-C-S-H addition, which was decreased from 157.0 mm of the paste with 0.2 wt.% sucrose addition to 154.0 mm, 151.0 mm, 148.0 mm and 137.0 mm respectively for the pastes with 0.5 wt.%, 1.0 wt.%, 2.0 wt.% and 4.0 wt.% nano-C-S-H gel incorporation.

In recent years, many studies have focused on the influences of the nanoparticle addition on fluidity or rheological behavior of the fresh cement pastes [50,51,54,55]. It has been indicated that the nanoparticles in either nanopowder or nanodispersion generally exist in an agglomerated (loosely-held clusters) or aggregated (firmly-held clusters) state with the final grain size from submicron to as high as 100 μm due to their very high specific surface area and energy [50,56,57,58]. Even the monodispersed nanoparticles in colloidal silica sol (CSS) coagulate immediately to form loose flocs due to the high ionic strength in cement pastes when cement is mixed with water containing CSS [51,59]. While incorporating colloidal boehmite, the nanoparticles may also aggregate or form clusters under the high ionic strength in fresh cement pastes [60,61]. Therefore, it should be the behavior of the final agglomerates, aggregates, flocs or clusters, rather than those individual nanoparticles, that controls the effects of them on properties of the fresh and hardened cement-based materials [51].

From the viewpoint of the filling effect, theoretically the nanoparticles help to improve fluidity of the paste if the agglomerates or aggregates introduced or the flocs immediately formed can function as fillers to occupy the void space among cement particles and release some free water in the space originally not contributing to fluidity, even though some free water in the space was adsorbed or constrained by them [50]. Otherwise, those agglomerates, aggregates or flocs unable to act as fillers will absorb or retain some free water in their voids thus the fluidity of the cement pastes will be reduced while incorporating nanomaterials under the condition that the water content was controlled nearly the same. Their impact is mainly determined by the water retention capacity and also the dosage of the nanomaterials. Typically, the immediately formed flocs while incorporating colloidal nanoparticles in the paste would possess a much higher water retention capacity than the agglomerates in nanopowder, whereas the agglomerates in nanopowder a higher capacity than the aggregates in nanodispersion. For the nanomaterials in the same dispersing state, the finer the primary nanoparticles are, the higher the water retention capacity is, because more water is required to coat and wet the nanoparticles.

However, it seemed that the nano-C-S-H gel used in this study possessed a much different characteristic from the other nanomaterials generally used in other studies. Though the C-S-H nanoparticles seemed to show obvious aggregation in the TEM photograph (Figure 2c), the testing results by using the laser diffraction analysis (Figure 2d) revealed that they were actually monodispersed in water and the particle size was only in the range of 0.5365–1.117 nm. For these monodispersed nanoparticles, the high ionic strength in the cement paste has no impact on their dispersing state because actually these nanoparticles were precipitated under a very high ionic strength during preparation. Without a further drying process, these nanoparticles can still be monodispersed in the cement pastes and typically are able to function as fillers to improve the fluidity of the fresh cement paste if not so much nano-C-S-H gel was incorporated. However, the fluidity of the fresh cement paste was still a little worsened even when a very small amount of nano-C-S-H gel was introduced (the C-S-H nanoparticles was 0.0263 wt.%, 0.0526 wt.%, 0.1052 wt.%, 0.2104 wt.% and 0.4208 wt.% of the cement respectively), as seen in Figure 7. The main reason why this happens was probably resulted from the fibrillar characteristic of the C-S-H nanoparticles (see Figure 2c). Due to the fibrillar morphology, the prepared nano C-S-H showed a gel-like substance even though the solid loading was only 5.26 wt.% (Figure 2a). Obviously incorporating these fibrillar nanoparticles will cause a reduced fluidity for the fresh cement-based materials. However, this gel-like substance can be redispersed in water very well and actually showed monodispersion as indicated by the laser diffraction analysis. It is not a real gel. They still show monodispersion without flocculation or gelation even under the very high ionic strength of the fresh cement paste. That might be the main reason why the flow value was not seriously reduced with increasing nano-C-S-H gel addition, as compared to the case that the CSS was incorporated into the cement paste, in which the flow value of the fresh mortar was reduced seriously with increasing CSS addition [51,59].

### 3.2. Cement Hydration

**Figure 8:**
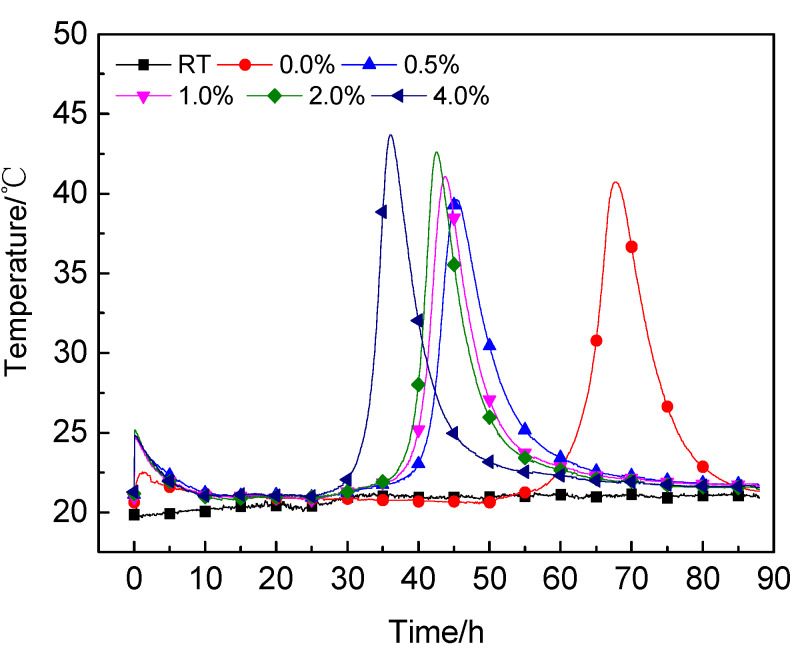
Effect of nano-C-S-H addition on cement hydration with 0.2 wt.% sucrose detected by semi-adiabatic calorimetry.

Figure 8 illustrates the influence of the nano-C-S-H gel addition on cement hydration with 0.2 wt.% sucrose as the retarder detected by the semi-adiabatic calorimetry. The testing results verified that incorporation of the nano-C-S-H gel greatly mitigated the retardation of cement hydration in the paste with sucrose as the retarder [18]. The time when the main peak of the temperature rise appeared was advanced from 67.7 h for the paste with only 0.2 wt.% sucrose to 45.3 h for the paste with 0.2 wt.% sucrose and 0.5 wt.% nano-C-S-H gel addition. With increasing nano-C-S-H gel addition, the time when the main exothermic peak appeared in the curve was further brought forward to 43.75 h, 42.58 h and 36.0 h respectively when the nano-C-S-H gel addition was increased to 1.0 wt.%, 2.0 wt.% and 4.0 wt.%.

In regard to the impact of different retarders on cement hydration, Bishop and Barronit [47] summarized four traditional inhibition mechanisms, including removing calcium from solution by calcium-chelating or forming insoluble salts to prevent C-S-H gel formation, inhibiting growth of C-S-H gel or Ca(OH)_2_ crystals by nucleation poisoning [42], surface adsorption of the retarder molecules directly onto the surface of either the anhydrous or (more likely) the partially hydrated mineral surfaces to block further reactions with water and formation of a semi-permeable layer on the cement grains to slow the migration of water and lengthen the induction period [62]. However, for the first mechanism, no simple correlation can be observed between either calcium binding strength or calcium salt solubility and retarding ability [48]. For the nucleation-poisoning mechanism, it seemed that it was also not necessarily true because the C-S-H gel may not form through nucleation and growth process [53]. According to the through-solution mechanism, many studies proposed that the nanoparticles act as nucleation sites for C-S-H growth during cement hydration. However, no nucleus function can be observed in the hydrating system with nano-silica and nano-TiO_2_ addition while using modeling experiments to investigate whether nanoparticles incorporated in the cement paste can act as nucleation sites or not for C-S-H growth during cement hydration.

**Figure 9:**
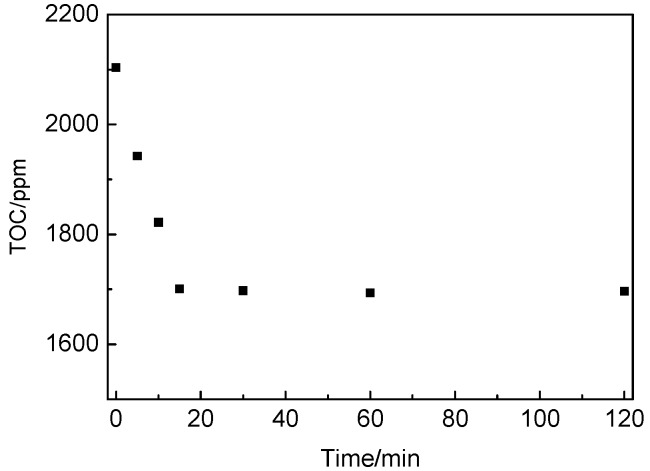
TOC content of the sucrose solution after nano-C-S-H gel were added for various time. (0.2 g sucrose dissolved in 36.2 g water, and then 4.0 g nano-C-S-H gel was added).

In the cement paste with sucrose addition, Ataie et al. [46] found that incorporation of supplementary cementitious materials (SCMs) such as rice straw ash (RSA), silica fume and metakaolin can also mitigate the retarding effect of the sucrose on cement hydration to a certain extent. The TOC of the solutions filtered from the pastes with both sucrose and SCM addition was clearly reduced though the SCMs themselves do not sorb sugar molecules in sucrose-NaOH solution. It was proposed that the impact of SCMs on sucrose retardation is resulted from the interaction between the sucrose and the products (probably the C-S-H gels) created by the SCMs in the system. Therefore, in this study, the interaction between the nano-C-S-H gel and the sucrose molecules was also investigated through the TOC measurement. The results are shown in Figure 9. As seen in Figure 9, the TOC of the filtrate from the nano-C-S-H dispersion in the sucrose solution decreased rapidly from the theoretical value of 2103.4 ppm at time zero to 1942.6 ppm, 1821.7 ppm and 1700.6 ppm after the nano-C-S-H gel was dispersed into the sucrose solution for 5 min, 10 min and 15 min, respectively. It revealed that the sucrose molecules could be rapidly absorbed by the nano-C-S-H gel within 15 min. At about 20 min, the absorption reached saturation and the saturated absorption ratio was about 100 ppm TOC/g gel.

**Figure 10:**
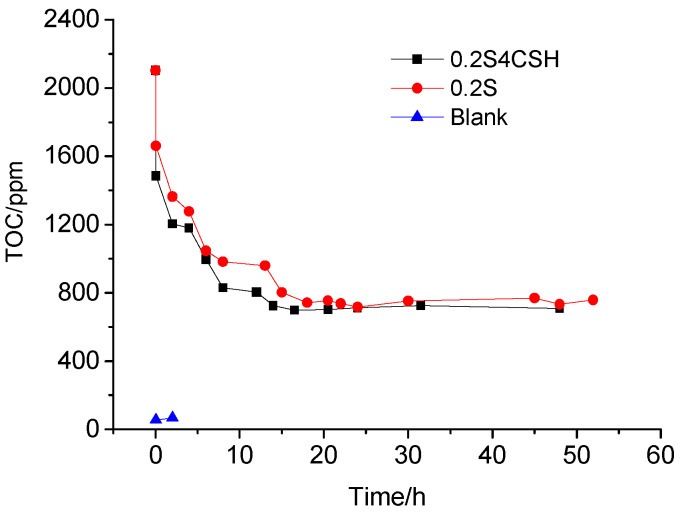
TOC content in pore solutions of the fresh cement pastes with a water cement ratio of 0.40 and with 0.2 wt.% sucrose and 4.0 wt.% nano-C-S-H gel addition.

Figure 10 illustrates the TOC contents of the solutions filtered from the cement pastes with and without sucrose and nano-C-S-H addition at various curing hours before the acceleration period. As seen in Figure 10, the TOC content in the pore solution from the blank cement paste was about 54.4 ppm and 67.0 ppm respectively at 3 min and 2 h of the curing age. The TOC in the blank paste may come from the tap water and the cement in case that no organic admixtures were introduced. In the paste with 0.2 wt.% sucrose addition the TOC content dropped sharply from the theoretical value of 2103.4 ppm at time zero to 1661.4 ppm after the cement was mixed into the sucrose solution for only 3 min. The main reason why the TOC content was reduced so much within the first several minutes was probably resulted from the adsorption of the sucrose molecules onto the wet cement particles. The absorption reached as high as 21.0%. Considering that some water was actually consumed to wet and react with the cement particles, the actual adsorption should be much higher than that calculated from the TOC testing results because the concentration calculated based on the TOC testing results was actually much higher than that calculated according to the water content used to prepare the cement pastes. After the paste was cured at room temperature, the TOC content decreased continuously to about 982.6 ppm after cured for 8 h. Then the TOC content showed a small decrease with increasing curing hours and was reduced to about 960.1 ppm at 13 h. However, it was further reduced with further increasing curing. The TOC content dropped to about 802.6 ppm and 742.5 ppm at 15 h and 18 h. After that, the TOC content seemed to stop decreasing and began to keep nearly the same at about 750 ppm until 52 h.

For the cement paste with both sucrose and nano-C-S-H gel addition, it can be seen from Figure 10 that the TOC content of the pore solution was also obviously reduced and lower than that of the pore solution from the paste with only sucrose addition. For example, the TOC content was only 1484.3 ppm and the adsorption reached as high as 29.4% after the cement was mixed with the water containing sucrose for 3 min. After being cured for 2 h and 8 h, the TOC content was reduced to about 1203.5 ppm and 830.0 ppm. For the paste cured from 8 to 12 h, the TOC content was further reduced, but only a little bit. However, at 14 h and 16.5 h, the TOC content was reduced a little more, but still less than that from the paste with only sucrose addition. After that, it seemed that the sucrose concentration did not show obvious reduction and began to keep nearly the same at about 710 ppm until 48 h. It revealed that addition of nano-C-S-H gel helped to reduce the TOC content in the pore solution by adsorption. However, the difference between them was only about 40 ppm.

According to the results illustrated in Figure 10, it seemed that the few changes in TOC content of the pore solution after 16 h might suggest the end of the induction period near this time during cement hydration, since sucrose was no longer adsorbed by the hydration products. However, the semi-adiabatic calorimetry showed that the induction period was much longer than 16 h, which lasted as long as 55 h for the paste containing 0.2 wt.% sucrose (Figure 8). While incorporating the nano-C-S-H gel, the induction period was greatly shortened, but was still longer than 16 h, as seen in Figure 8. The few changes in TOC after 16 h could be explained by the hydration products that cannot absorb sucrose molecules anymore because no more new hydration products were formed after this time. The reason why the induction period lasts such a long time for the paste with 0.2 wt.% sucrose addition was probably caused by the reduced permeability of the protective layers when the sucrose molecules were absorbed into them. For the paste with both sucrose and nano-C-S-H gel addition, typically fewer sucrose molecules were absorbed into the protective layers because some of them were absorbed by the C-S-H nanoparticles before their formation. Therefore, the permeability of the protective layers was reduced less, thus the retarding effect of the sucrose on cement hydration was mitigated.

However, it seemed that a new problem appeared: why can not the sucrose molecules be further absorbed into the protective layers to reduce their permeability thus further retarding the cement hydration since the TOC content was quite high in the pore solution from the paste with both sucrose and nano-C-S-H gel addition? In the paste with only sucrose addition, it can be easily explained by the saturation absorption of the sucrose molecules, but in the paste with both sucrose and nano-C-S-H gel addition, it is hard to imagine by the saturation absorption any more, because if the adsorption reached saturation, the permeability of the protective layers should be nearly the same as that without the nano-C-S-H gel and the addition of nano-C-S-H gel should not be able to mitigate the retardation. The fact that the addition of nano-C-S-H gel can effectively accelerate the retarded cement hydration indicated that the permeability of the protective layer must be improved by the nano-C-S-H gel addition. Therefore, the reason why the pore solution still showed a pretty high TOC content was probably resulted from that the organic carbon in the pore solution actually exists as sucrose half-salt [42], which cannot be adsorbed into the protective layer. That might be the main reason why the addition of nano-C-S-H gel helped to greatly mitigate the retardation caused by the addition of sucrose in the cement paste.

### 3.3. Hydration Products

**Figure 11:**
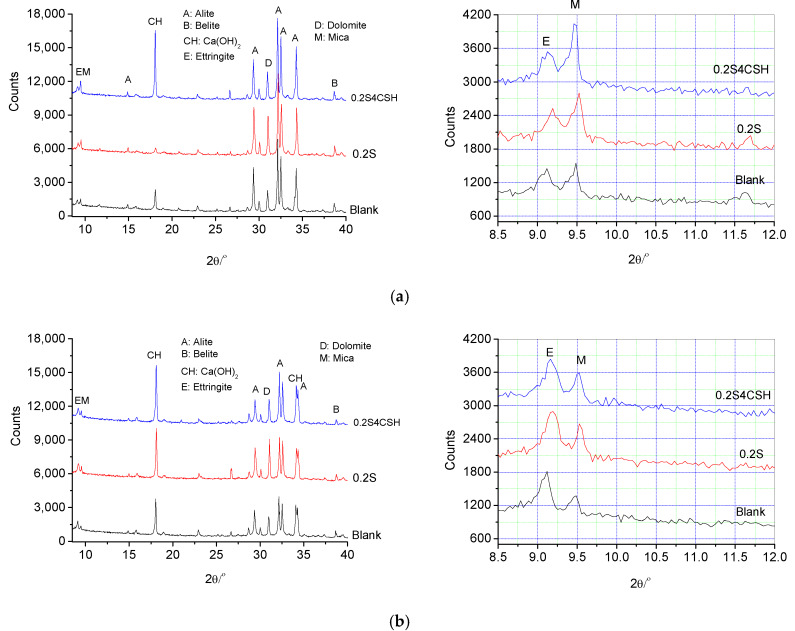

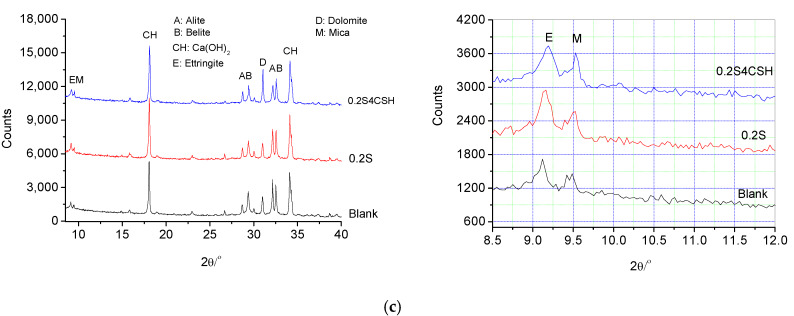
XRD diagrams of the hardened cement paste (HCP) with and without sucrose (0.2 wt.%) and the nano-C-S-H gel (4.0 wt.%) addition at (**a**) T1, (**b**) T2, (**c**) T3.

Figure 11 illustrates XRD patterns of the fresh and hardened cement pastes with 0.2 wt.% sucrose together with and without 4.0 wt.% C-S-H gel addition at T1, T2 and T3, i.e., the time when the induction period ends, the time when the main exothermic peak appears and the time when the deceleration period ends, respectively, in the temperature rise curves. From Figure 11a, it can be seen that at T1 the calcium hydroxide (CH) has begun to crystallize in the cement paste without sucrose addition. However, in the paste with 0.2 wt.% sucrose addition, few CH crystals can be observed according to the XRD analysis. About the reason why much fewer CH formed at T1 in the paste with 0.2 wt.% sucrose addition, it seemed that it can be explained by the much lower permeability of the protective layers after the sucrose molecules were absorbed onto the hydrating cement particles, inhibiting calcium ions diffused into the external solution in the cement paste. However, the analysis of the aqueous phase of hydrating cement paste by Thomas et al. [42] revealed increases in the concentration of Ca^2+^, OH^−^, Si, Al and Fe in the presence of sucrose. If so, the probable explanation may be that these increases happen before the formation of the protective layers because the calcium ions cannot diffuse into the external solution through the semi-permeable C-S-H protective layers once they formed. From this viewpoint, the reason why few CH crystals formed may really be resulted from the so-called poisoned nucleation of the CH crystals due to the existence of the sucrose in the paste. Even though more calcium ions were released, they were combined or chelated by sucrose molecules, thus no nucleus formed for crystallization of the CH after the protective layers formed.

However, for the paste with both 0.2 wt.% sucrose and 4.0 wt.% C-S-H gel addition, it is interestingly found that the main diffraction peak of CH at about 18° of 2θ was much higher than that of the blank and the paste with 0.2 wt.% sucrose addition. It indicated that the incorporation of the nano-C-S-H gel into the paste with sucrose addition could greatly improve the permeability of the protective layer around the hydrating cement particles. However, it seemed that the CH crystals were more prone to grow along the (0001) plane with a larger size in the paste with nano-C-S-H gel addition, as also observed in the cement paste with colloidal silica sol addition [51]. It implied that the nano-C-S-H gel in the paste can also form a 3D network to slow down the diffusion rate of the released ions and eliminate the convection in the paste, thus the 3D nucleation and growth of the CH crystals were suppressed, as discussed in the previous study [51]. As seen in Figure 11b, at T2, the diffraction peak of the CH at about 18° of 2θ was clearly improved for the blank paste as compared to that at T1. For the paste with 0.2 wt.% sucrose addition, the peak of the calcium hydroxide at about 18° of 2θ was also greatly enhanced as compared to that at T1, and it was even a little higher than that in the blank paste. While further incorporating the nano-C-S-H gel in the paste with 0.2 wt.% sucrose, the peak at T2 was a little lower than that at T1, but it was still a little higher than that in the blank paste and the paste with only 0.2 wt.% sucrose addition. It verified the sucrose as a “delayed accelerator” [63] to accelerate the cement hydration between T1 and T2 and further incorporation of the nano-C-S-H gel can further accelerate the cement hydration.

From Figure 11c, it can be seen that the diffraction peaks of CH in the cement pastes with and without sucrose and the nano-C-S-H gel were further improved and those main peaks corresponding to the alite were further reduced at T3 while comparing to those at T2. It revealed that the cement particles continue to hydrate in a high speed even in the deceleration period. More alite was consumed and more CH was produced. At the same time, it can be seen that more alite was consumed in the paste with 0.2 wt.% sucrose addition as compared to the blank paste, and while incorporating nano-C-S-H gels to promote the cement hydration by sucrose as a retarder, more alite was also consumed than that without nano-C-S-H gel addition.

While enlarging the AFt peaks in the XRD curves, it can be seen that addition of sucrose seemed to cause a refinement of the ettringite crystals during cement hydration at T1, T2 and T3, as also indicated by Bishop et al. [47], but further incorporation of the nano-C-S-H gel showed no further or even a reverse effect. As seen in Figure 11, the diffraction peak of the ettringite at about 9.125 of 2θ showed a little wider for the HCP with 0.2 wt.% sucrose addition, as compared to that of the blank, especially at T2 and T3. For the paste with both sucrose and nano-C-S-H addition, it was a little narrower than that in the paste with only sucrose addition, but still a little wider than that in the blank paste. The fact that the diffraction peak showed a little wider indicates a refinement of the ettringite crystals in the paste with sucrose addition. However, the crystals were coarsened a little bit when the nano-C-S-H gel was incorporated into the retarded cement paste.

**Figure 12:**
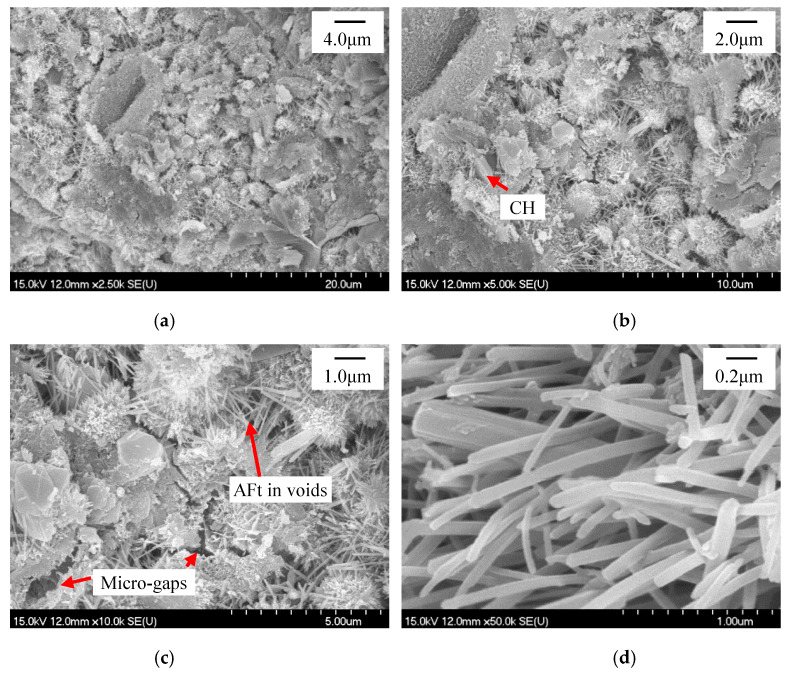

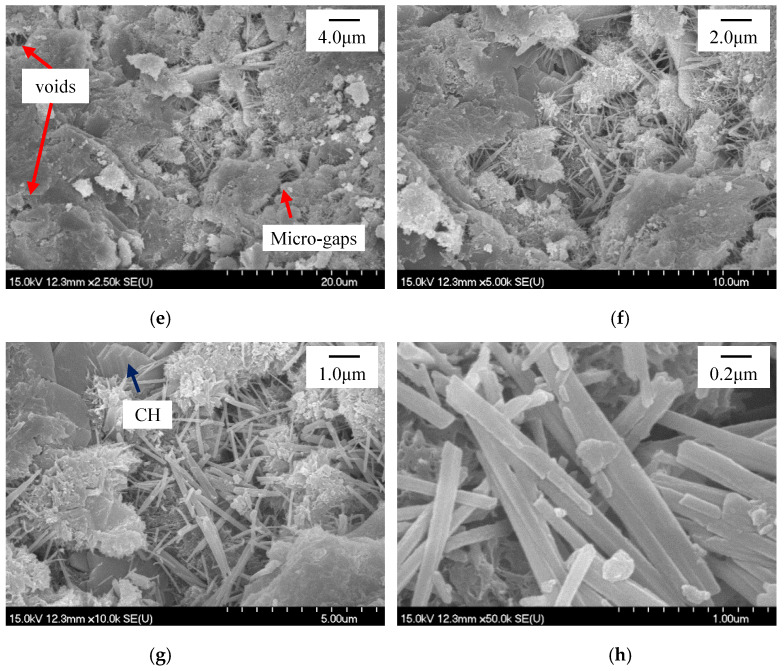
SEM photographs for the blank HCP at T2 and T3. (**a**)–(**d**) T2, (**e**)–(**h**) T3.

**Figure 13:**
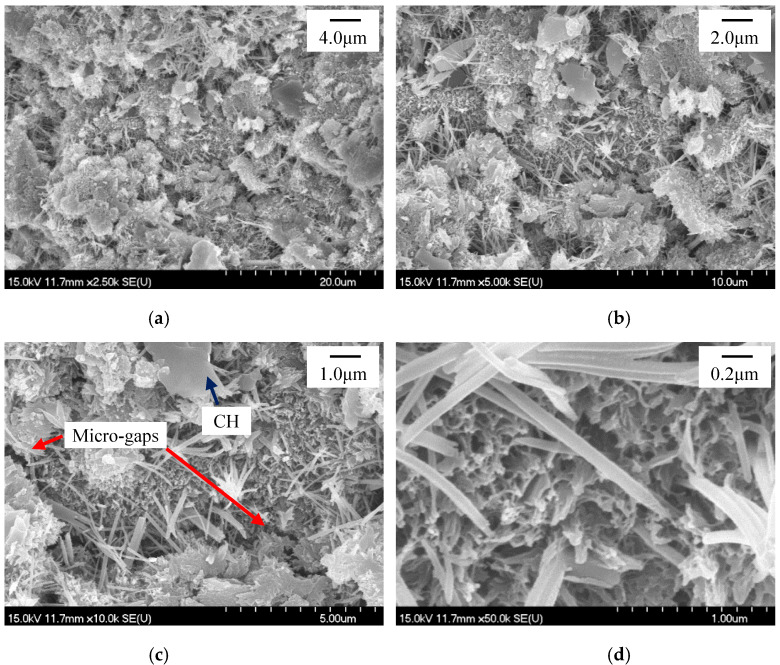

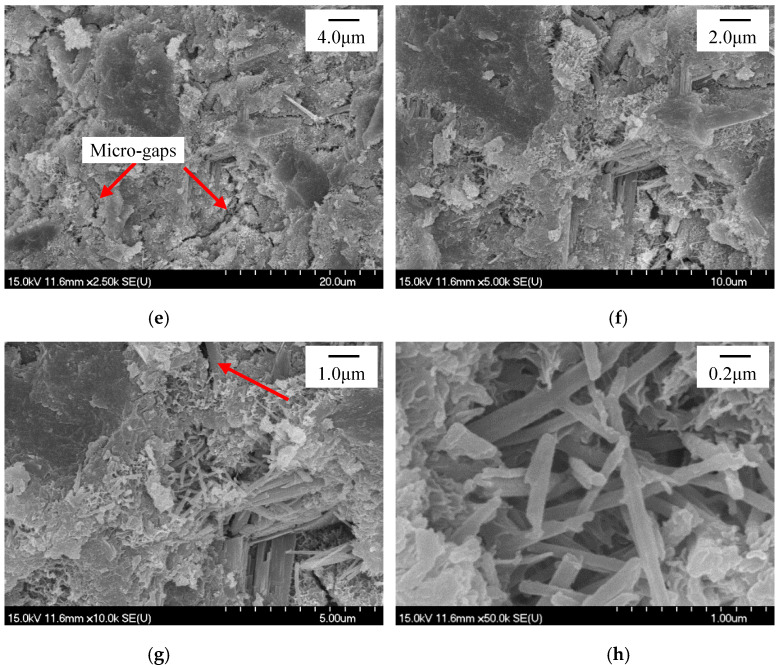
SEM photographs for the HCP with 0.2 wt.% Sucrose at T2 and T3. (**a**)–(**d**) T2, (**e**)–(**h**) T3.

**Figure 14:**
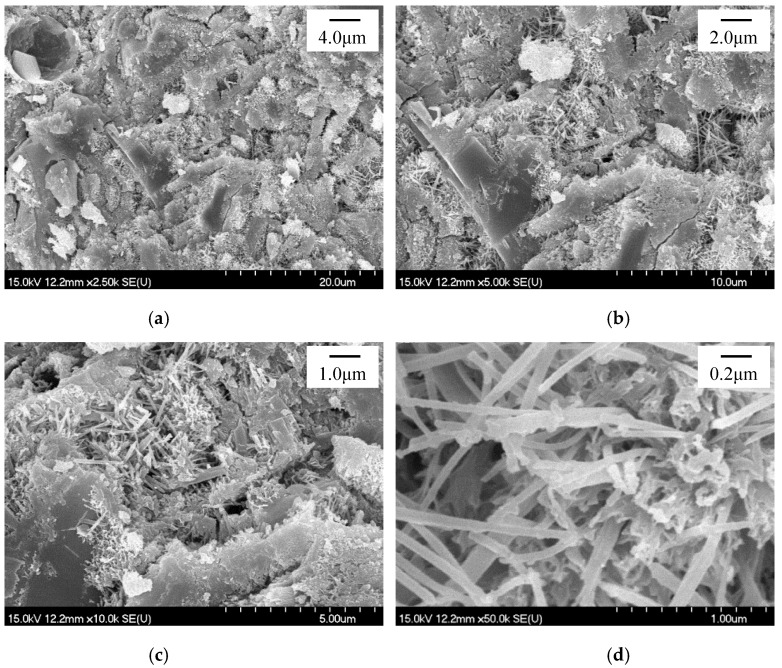

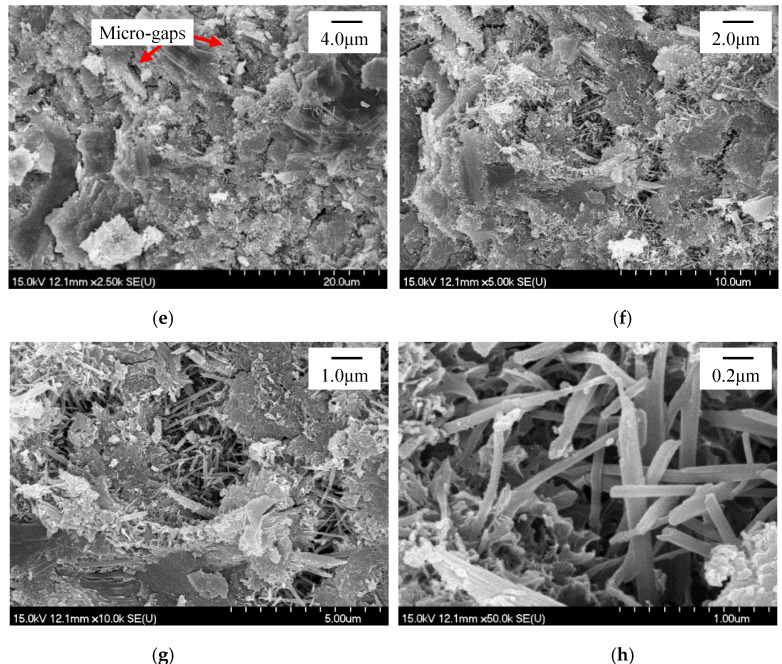
SEM photograph for the HCP with 0.2 wt.% Sucrose and 4.0 wt.% nano-C-S-H at T2 and T3. (**a**–**d**) T2, (**e**–**h**) T3.

The refinement of the ettringite crystals in the HCP with sucrose addition was also verified by the SEM observation. At the same time, it seemed that the microstructure of the HCP was also improved while incorporating sucrose as the retarder, and further improvement was observed while further dosing nano-C-S-H gel according to the SEM observation. Figure 12, Figure 13 and Figure 14 present the SEM photographs of the HCP with and without sucrose and nano-C-S-H gel addition at T2 and T3. As seen in Figure 12a–c, the blank HCP didn’t compact very much at T2. Though the hydration products contact with each other, resulting in setting and hardening of the cement paste, there still exist obvious voids and micro-gaps among the hydrated cement particles and the ettringite crystals packed together loosely in the voids. From Figure 12a–c and Figure 12e–g, it can be seen that the blank HCP showed a typically higher compaction at T3 than that at T2, illustrating that the hydration products still formed continuously in the deceleration period.

While incorporating 0.2 wt.% sucrose, it seemed that the microstructure of the HCP (Figure 13a–c,e–g) was improved a little bit at both T2 and T3 as compared to the blank HCP (Figure 12a–c,e–g). Though voids and micro-gaps among the hydrated cement particles can still be observed (Figure 13c), they were reduced and narrowed to a certain extent in the HCP with 0.2% sucrose addition as compared to those in the blank HCP (Figure 12c). The loosely packed ettringite crystals can be easily detected in the voids too (Figure 13d,h). However, they were refined a little bit, as compared between Figure 12d,h and Figure 13d,h.

While further introducing the nano-C-S-H gel into the paste with 0.2 wt.% sucrose addition, the microstructure seemed to be further improved, as compared between Figure 13a,e and Figure 14a,e. The voids and micro-gaps were further reduced and narrowed for the HCP with 0.2 wt.% and further with 4.0 wt.% nano-C-S-H gel addition, as compared to that with only 0.2 wt.% sucrose addition. However, the ettringite crystals were not further refined, and even coarsened a little bit while incorporating the nano-C-S-H gel into the cement paste with sucrose addition, as compared between Figure 13d and Figure 14d or between Figure 13h and Figure 14h. The improvement of the microstructure of the HCP and the refinement of the ettringite crystals may contribute to the improvement in mechanical properties of HCP and also the hardened cement-based materials [64], which will be reported in a companion paper in the future.

## 4. Conclusions

Based on the experimental results, the following conclusions can be drawn:

- The TEM observation revealed that the chemically synthesized C-S-H gel was fibrous in the nanoscale. Due to its fibrillar morphology, the C-S-H showed a gel-like substance even though the solid loading was only 5.26 wt.%. However, the laser diffraction analysis revealed that the nano-C-S-H gel could be re-dispersed in water very well. They were monodispersed without flocculation or gelation even under the very high ionic strength during preparation. Therefore, the flow value of the fresh cement paste was gradually but slightly reduced with increasing nano-C-S-H gel addition.
- The semi-adiabatic calorimetry testing results verified an obvious mitigation of the serious retarding effect of the sucrose on cement hydration by incorporating the nano-C-S-H gel. The total organic carbon (TOC) analysis revealed a rapid adsorption of the sucrose molecules by the C-S-H nanoparticle and a little lower TOC content in the pore solutions of the cement paste with both sucrose and nano-C-S-H gel addition than that with only sucrose. However, in both retarded pastes with and without the nano-C-S-H gel, the TOC content in the pore solutions still reached as high as about 800 ppm after it stopped decreasing at about 16 h, which is far before the induction period starts in the paste with sucrose addition. It was proposed that the sucrose in the cement paste exists as half-salt, which cannot be absorbed into the protective layer to further reduce the permeability. Thus, the addition of the nano-C-S-H gel can help to reduce the adsorption of the sucrose molecules into the protective layer and greatly mitigate the retardation caused by the sucrose.
- Through XRD analysis, it was found that before the induction period starts the CH crystals are more prone to grow along the (0001) plane with a larger size in the paste with the nano-C-S-H gel addition, which can form the 3D network to slow down the diffusion rate of the released ions and eliminate the convection in the paste, thus suppressing the 3D nucleation and growth of the CH crystals. At the same time, the XRD analysis also indicated a refinement of the ettringite crystals in the cement paste with sucrose addition and the further introduction of nano-C-S-H gel did not show further refinement or even a reverse effect, as also verified by the SEM observation.

## Figures and Tables

**Table 1:** Chemical compositions of P.W 52.5 cement.

Component	CaO	SiO_2_	MgO	SO_3_	Al_2_O_3_	Na_2_O	K_2_O	Fe_2_O_3_	TiO_2_
Wt/%	64.76	22.04	6.02	2.93	2.49	0.713	0.564	0.272	0.06

**Table 2:** Physical and mechanical properties of P.W 52.5 cement.

Setting Time/min		Flexural Strength/MPa		Compressive Strength/MPa			
Initial condensation	Final condensation	3 d	28 d		3 d	28 d	
157	209	6.9	8.8		39.9	57.4	

## Abbreviations

Abbreviation	Full Name
C-S-H	calcium silicate hydrate
TOC	total organic carbon
XRD	X-ray diffraction analysis
CH	calcium hydroxide
SEM	scanning electron microscope
HPC	high-performance concrete
HCP	hardened cement paste
ITZ	interfacial transition zone
XRF	X-ray fluorescence analysis
TDS	total dissolved solids
TEM	Transmission electron microscope
CSS	colloidal silica sol
C/S	CaO:SiO_2_
C_3_S	tricalcium silicate
SCMs	supplementary cementitious materials
RSA	rice straw ash
T1	the time when the induction period ends and the acceleration starts
T2	the time when the exothermic peak appears
T3	the time when the deceleration period ends
